# Delayed convergence of iterative reconstruction in dopamine transporter SPECT with multiple-pinhole collimators^☆^

**DOI:** 10.1016/j.zemedi.2025.10.006

**Published:** 2025-11-05

**Authors:** Loreen Jedid, Thomas Buddenkotte, Ivayla Apostolova, Laurin Bruer, Erik Jámbor, Susanne Klutmann, Melinda Szolikova, Attila Forgács, Svend Hvidsten, Ralph Buchert

**Affiliations:** a Department of Diagnostic and Interventional Radiology and Nuclear Medicine, University Medical Center Hamburg-Eppendorf, Martinistr. 52, Hamburg 20246, Germany; b Mediso Ltd., Laborc utca 3, Budapest H-1037, Hungary; c Department of Nuclear Medicine, Odense University Hospital, J. B. Winsløws Vej 4, Odense C 5000, Denmark

**Keywords:** SPECT, collimator, multiple-pinhole, iterative reconstruction, convergence, dopamine transporter

## Abstract

**Background:**

Multiple-pinhole (MPH) collimators for brain SPECT improve the resolution-sensitivity tradeoff compared with conventional parallel-hole and fan-beam collimators. The system count sensitivity profile with MPH collimators exhibits a peak at the center of the field-of-view and significant decline in count sensitivity toward the periphery. In MPH SPECT with [^123^I]FP-CIT, this makes the difference in relative statistical noise between the striatum and extrastriatal (reference) regions even bigger. This could slow down the convergence of iterative reconstruction compared with [^123^I]FP-CIT using conventional collimators. This study evaluated the impact of the effective number of reconstruction iterations in [^123^I]FP-CIT SPECT with MPH collimators.

**Methods:**

671 patients (43.8% females, 67.3±11.3y) were included retrospectively. Projection data were acquired with a triple-head camera (Mediso AnyScan Trio) equipped with 2^nd^ generation general-purpose brain MPH collimators. MPH projections from 12 min total scan duration were reconstructed with the iterative 3d Tera-Tomo Monte Carlo algorithm of the system software. The effective number of iterations was varied between 12 and 300. To evaluate the impact of the iteration number on diagnostic performance, a data-driven Gaussian mixture model approach was used to assess the power of the putaminal specific binding ratio (SBR) for differentiation between reduced and normal scans. In a subset of 72 patients, reconstructed images were visually evaluated twice by 7 independent readers with respect to parkinson-like reduction of striatal [^123^I]FP-CIT uptake using a Likert 6-score (-3=clearly reduced, …, 3=clearly normal). The impact of the iteration number on the Likert 6-score was tested using repeated measure analysis of variance (ANOVA) with iteration number, reader and session as within-subject factors. The majority vote binary classification (reduced, normal) was taken into account as between-subject factor. Within- and between-readers agreement of visual scoring were characterized by Cohen’s and Fleiss’ kappa.

**Results:**

Iterative reconstruction converged much slower in the extrastriatal reference region than in the striatum and its subregions. Considerable improvement of spatial resolution with increasing iteration number came with an acceptable increase of statistical noise. The discriminative power of the putaminal SBR was highest with 300 effective iterations. There was a small but significant reduction of reader confidence and between-readers agreement with 300 compared to 24 iterations, particularly in normal cases (Likert 6-score 2.325 versus 2.468, p < 0.001).

**Conclusions:**

300 iterations provide a good compromise between spatial resolution, robustness and diagnostic power of semi-quantitative SBR analyses on one side versus statistical noise and between-readers variability on the other side. In order to avoid relevant loss of between-readers stability, readers should be specifically trained for reading [^123^I]FP-CIT MPH SPECT images reconstructed with a high number of iterations.

## 1 Introduction

Single-photon emission computed tomography (SPECT) using the dopamine transporter (DAT) ligand N-ω-fluoropropyl-2β-carbomethoxy-3β-(4-I-123-iodophenyl)nortropane ([^123^I]FP-CIT) is commonly used to aid in the etiological diagnosis of uncertain parkinsonian syndromes and to distinguish dementia with Lewy bodies from Alzheimer’s disease [[1], [2], [3], [4], [5]].

Multiple-pinhole (MPH) collimators concurrently improve both spatial resolution and system count sensitivity compared to parallel-hole and fan-beam collimators [[6], [7], [8], [9], [10], [11], [12], [13], [14]]. Due to the non-uniformity of system count sensitivity with MPH collimators across the acquisition field-of-view (FOV), only iterative methods are suitable for reconstructing tomographic SPECT images from MPH projection data [14,15].

The rate of convergence of iterative reconstruction varies within the FOV, depending on the size of the considered objects and the regional level of statistical noise (“the slower, the noisier”) [[16], [17], [18]]. Thus, the number of iterations required to avoid systematic errors due to incomplete convergence of iterative reconstruction strongly depends on the low-count regions of interest. In [^123^I]FP-CIT SPECT, these regions include extrastriatal brain areas that are used as a reference for striatal tracer uptake in both quantitative and visual evaluations.

The system count sensitivity profile with MPH collimators exhibits a peak at the center of the FOV (where the striatum is positioned in [^123^I]FP-CIT SPECT) and a significant decline in count sensitivity toward the periphery of the FOV (where extrastriatal brain regions are located) [14,19,20] (Supplementary Fig. 1). Thus, the *difference* in the relative statistical noise levels between the striatum and extrastriatal reference regions can be considerably larger in MPH SPECT than in SPECT with parallel-hole collimators [21]. The latter provide uniform system count sensitivity throughout the entire FOV. This could result in slower convergence of iterative reconstruction in extrastriatal regions in [^123^I]FP-CIT SPECT with MPH collimators requiring a higher number of iterations to avoid incomplete convergence compared to parallel-hole collimators.

Against this background, the current study aimed to analyze the impact of the effective number of iterations in iterative Monte Carlo reconstruction in [^123^I]FP-CIT SPECT using a triple-head SPECT camera with MPH collimators for brain imaging.

## 2 Materials and Methods

### 2.1 Patients

This retrospective study included 671 consecutive patients (43.8% female, 67.3 ± 11.3y, range 26.4–91.6y) who were referred for [^123^I]FP-CIT SPECT imaging in the Department of Nuclear Medicine at the University Medical Center Hamburg-Eppendorf due to a clinically uncertain parkinsonian syndrome or suspected dementia with Lewy bodies. No eligibility criteria were applied to ensure that the included patient sample was representative of clinical routine. The [^123^I]FP-CIT SPECT scans were acquired between 02/2020 and 04/2022.

Waiver of informed consent for the retrospective analysis of the anonymized data was obtained from the ethics review board of the general medical council of the state of Hamburg, Germany. All procedures were in accordance with the ethical standards of the ethics review board and with the 1964 Helsinki declaration and its later amendments.

Most of the patients were previously included in a study on the scan duration in [^123^I]FP-CIT SPECT with MPH collimators [22].

### 2.2 MPH SPECT

SPECT acquisition in list mode started 3.4 ± 0.4 h after intravenous injection of 181 ± 12 MBq [^123^I]FP-CIT. A total of 90 projection views (30 per head, 120° scan arc) with angular steps of 4° were acquired in list mode using a triple-head general-purpose camera (AnyScan Trio S, Mediso Medical Imaging Systems, Budapest, Hungary) equipped with second-generation general-purpose brain MPH collimators [19]. The acquisition time per projection was 60 s, resulting in a total net scan duration of 30 min. The energy window was set to 143-175 keV. The distance between the center-of-rotation axis and the pinhole focal plane was fixed at 150 mm. Helical acquisition mode with 40 mm total table displacement was used to avoid axial undersampling [23,24].

Projection data corresponding to 12 min total net scan duration were obtained by restricting the recorded events to the first 24 s for each projection angle [22]. The restricted projection data were sorted into 256x256 matrices with 2.13x2.13 mm^2^ pixel size.

Transaxial images were reconstructed using the Tera-Tomo Monte Carlo photon simulation engine with an iterative one-step-late maximum-a-posteriori expectation-maximization algorithm implemented in the camera software. The effective number of iterations was varied from 12 to 300. A detailed description of the tested reconstruction settings is provided in Table 1.

**Table 1:** Reconstruction settings of the 3d Tera-Tomo Monte Carlo reconstruction implemented in the SPECT system software (AnyScan Trio S, Mediso Medical Imaging Systems. Prefilter: none, regularization: adaptive bilateral medium, Monte Carlo quality: low, decay correction: yes, postfilter: none).

	it-12	it-24	it-54	it-99	it-198	it-300
# Effective iterations	12	24	54	99	198	300
# Subsets	2	2	3	3	3	3
Matrix	128x128	128x128	128x128	128x128	64x64	64x64
Voxel size [mm^3^]	1.8x1.8x1.8	1.8x1.8x1.8	1.8x1.8x1.8	1.8x1.8x1.8	3.6x3.6x3.6	3.6x3.6x3.6

Correction for photon attenuation and scatter was not performed because it most likely does not affect the ability of [^123^I]FP-CIT SPECT to distinguish between normal striatal signal and parkinson-like reduction [25].

### 2.3 Image preprocessing

Individual [^123^I]FP-CIT SPECT images were spatially normalized to the anatomical space of the Montreal Neurological Institute (MNI) using the Normalize tool of the Statistical Parametric Mapping software package (version SPM12) and a set of custom [^123^I]FP-CIT SPECT templates representative of normal and different levels of parkinson-like reduction of striatal uptake as target [26].

**Fig. 1:**
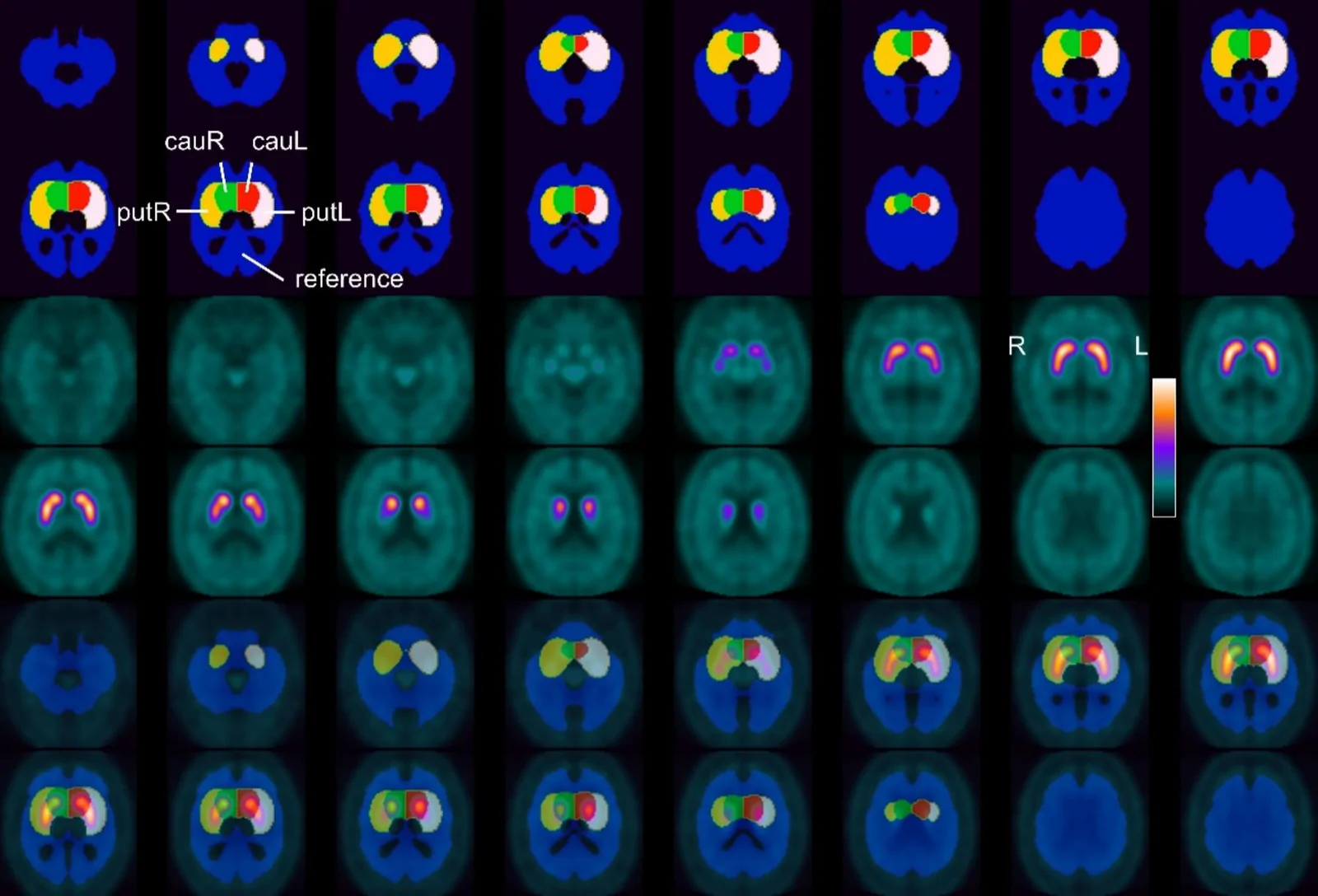
**Top two rows:** Region-of-interest masks of right (R) and left (L) caudate (cau; green/red) and putamen (put; yellow/white) used for hottest voxels analysis, and region-of-interest mask of the reference region (blue). **Middle two rows:** [^123^I]FP-CIT template. **Bottom two rows:** region-of-interest masks overlaid to the template. The region-of-interest masks and the template were predefined in the anatomical space of the Montreal Neurological Institute.

Intensity normalization was achieved by voxelwise scaling to the individual 75^th^ percentile of the voxel intensity in a reference region comprising the whole brain without striata, thalamus, brainstem, cerebellum, and ventricles [27] (Fig. 1). The scaled images are semi-quantitative images representing the distribution volume ratio (DVR).

### 2.4 Semi-quantitative analysis

The specific binding ratio (SBR) of [^123^I]FP-CIT in left and right striatum and its subregions (caudate nucleus, putamen) was obtained by hottest voxels analysis of the spatially normalized DVR image using large 3-dimensional unilateral region-of-interest (ROI) masks predefined in MNI space [28]. The ROI masks were much bigger than the actual anatomical structures in order to guarantee that all counts from the structure were included (Fig. 1). The number of hottest voxels to be averaged within a unilateral ROI mask was fixed to a total volume of 15 ml, 5 ml and 10 ml for striatum, caudate nucleus and putamen, respectively. SBR estimates were computed as mean DVR in the hottest voxels minus 1.

### 2.5 Visual reading

Visual reading of the [^123^I]FP-CIT SPECT images was based on a 12 mm thick DVR slab obtained by averaging 6 transversal slices of 2 mm thickness through the striatum from the spatially normalized DVR image, as described previously [29,30]. A fixed calibration of the color scale was used for standardized display [29,30]. The calibration of the color scale was fixed separately for each reconstruction setting. Representative examples are shown in Supplementary Fig. 2.

Visual reading of the slab images was performed independently by 7 readers blinded for all clinical information and for semi-quantitative estimates. The readers were asked to score each slab image regarding the presence of parkinson-like reduction of the striatal [^123^I]FP-CIT uptake using the following Likert 6-score: -3 = clearly reduced, -2 = probably reduced, -1 = more likely reduced than normal, 1 = more likely normal than reduced, 2 = probably normal, 3 = clearly normal. The Likert 6-score was intended to characterize the readers’ confidence in the binary classification of a case as either “reduced” or “normal”. The score was not intended for semi-quantitative grading of the striatal signal (e.g., to characterize the severity of nigrostriatal degeneration). Thus, the readers were asked to score a clear loss of the [^123^I]FP-CIT signal restricted to the posterior putamen in one hemisphere as -3 = clearly reduced (Supplementary Fig. 3). In addition, the readers were asked to score a rather uniform reduction of the striatal signal with clear delineation of the complete bilateral striatum as 3 = clearly normal [31,32] (Supplementary Fig. 4). Finally, the readers were asked for “conservative” reading by favoring specificity over sensitivity [31]. Thus, borderline cases should have been scored as 1 (more likely normal than reduced) rather than -1 (more likely reduced than normal) (Supplementary Fig. 5).

Visual reading was restricted to 72 of the 671 cases and to two reconstruction settings: it-24 and it-300. All except one of the 72 cases were used previously in a prospective study on the impact of MPH versus low-energy-high-resolution-high-sensitivity collimators on visual interpretation of [^123^I]FP-CIT SPECT [19]. The two reconstruction settings were selected based on an assessment by a highly experienced reader (> 20 years, > 5,000 cases). This reader was not among the 7 readers who performed the visual reading for the analyses reported here.

Visual reading was performed twice for each of the two settings, that is, each reader performed 4 reading sessions. All reading sessions were performed consecutively by each reader without washout phase between sessions. A different randomization of the images was used in each session. The reading sessions were grouped according to the reconstruction setting, that is, the two it-24 sessions were performed first followed by the two it-300 sessions, or vice versa. The ordering of the reconstruction settings was randomized across the 7 readers.

The 7 readers included one nuclear medicine physician with approximately 15 years of experience in the clinical reading of [^123^I]FP-CIT SPECT (≈ 3,000 cases) and 6 medical students in the third year of study without any prior experience in this task.

In preparation of the visual reading for the current study, all 7 readers underwent a joint 60 minutes training (including Supplementary Figs. 2-5) by an experienced reader (20 years, ≥ 5,000 cases). The training was concluded with 20 training cases (Supplementary Fig. 2).

### 2.6 Statistical analysis

The impact of the reconstruction setting on the power of the putamen SBR to discriminate between reduced and normal cases was tested using a Gaussian mixture model approach as described previously [22,25]. In brief, the distribution of the putamen SBR (minimum of both hemispheres, n=671) was characterized by a histogram with bin width of 0.1. The resulting histogram was fitted by the sum of two Gaussians:

(1) $$histogram\left(SBR\right)=A_{1}exp\left(-\frac{{\left(SBR-M_{1}\right)}^{2}}{{2SD}_{1}^{2}}\right)+A_{2}exp\left(-\frac{{\left(SBR-M_{2}\right)}^{2}}{{2SD}_{2}^{2}}\right)$$

where *A_1_*, *A_2_* are the amplitudes, *M_1_*, *M_2_* are the mean values and *SD_1_*, *SD_2_* are the standard deviations of the Gaussian functions. The MATLAB routine ‘fminsearch’ with default parameter settings was used for this purpose.

The discriminative power of the putamen SBR was estimated by the effect size *d* of the distance between the two Gaussians computed as the differences between their mean values scaled to the pooled standard deviation

(2) $$d=\left(M_{2}-M_{1}\right)/\sqrt{\frac{{\mathrm{SD}}_{1}^{2}+{\mathrm{SD}}_{2}^{2}}{2}}$$

The histogram analysis was performed separately for each reconstruction setting.

The majority vote binary classification (normal or reduced) of the 72 cases included in the visual reading was obtained by averaging the Likert 6-score across all readers (n = 7) and both reading sessions. Cases with mean Likert 6-score ≥ 0 were considered normal, cases with mean Likert 6-score < 0 were considered reduced. This was performed separately for both reconstruction settings included in the visual reading (it-24, it-300).

The impact of the reconstruction setting on the visual Likert 6-score was tested using repeated measure analysis of variance (ANOVA) with setting (it-24, it-300), reader (n = 7) and session (n = 2) as within-subject factors. The majority vote binary classification (reduced, normal) was taken into account as between-subjects factor.

Linearly weighted Cohen’s kappa and (unweighted) Fleiss kappa were used to characterize within- and between-readers variability of the Likert 6-score and of the binary visual interpretation derived from the Likert 6-score (negative 6-score -> reduced, positive 6-score -> normal). This was done separately for both reconstruction settings. Statistical significance of kappa differences between the two reconstruction settings was tested by checking their 83.4% confidence intervals (CI) for overlap, with non-overlapping 83.4%-CI indicating statistical significance with 5% type 1 error probability [33]. The 83.4%-CI was computed as: kappa ± 1.385* standard error of kappa [34]. The interpretation of Fleiss kappa values regarding the strength of within- and between-raters agreement was according to Landis and Koch [35].

Statistical analyses were conducted with MATLAB (version 2017b).

## 3 Results

### 3.1 Representative cases

**Fig. 2:**
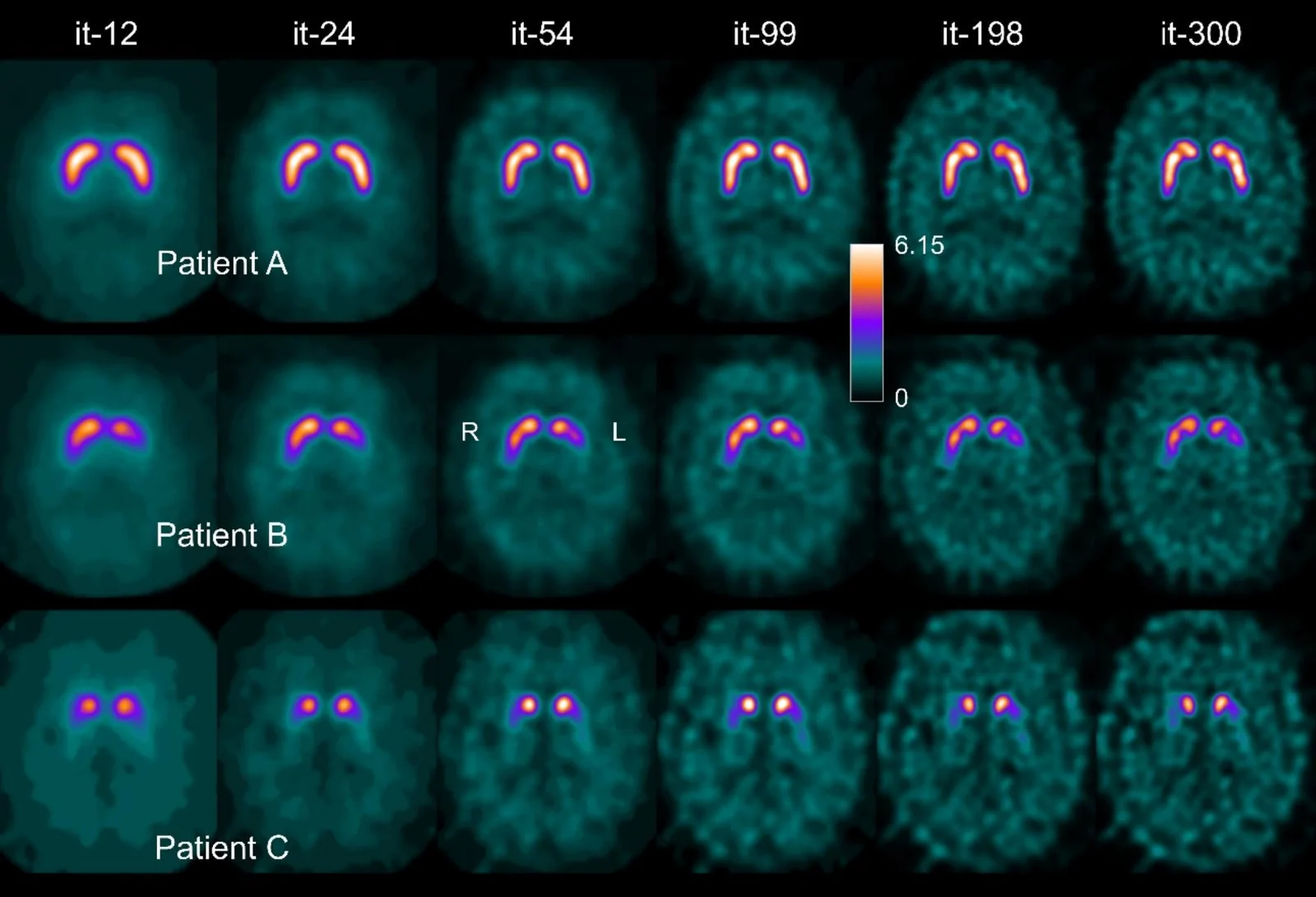
Two-dimensional slab view images of three patients with normal (top), moderately reduced (middle) and strongly reduced (bottom) putaminal [^123^I]FP-CIT uptake. The slab view images were obtained from 3-dimensional images reconstructed with 12, 24, 54, 99, 198 and 300 effective iterations (Table 1).

Two-dimensional slab view images obtained with the different reconstruction settings are shown in Fig. 2 for three representative cases. Increasing the number of effective iterations resulted in higher spatial resolution and higher noise levels.

**Fig. 3:**
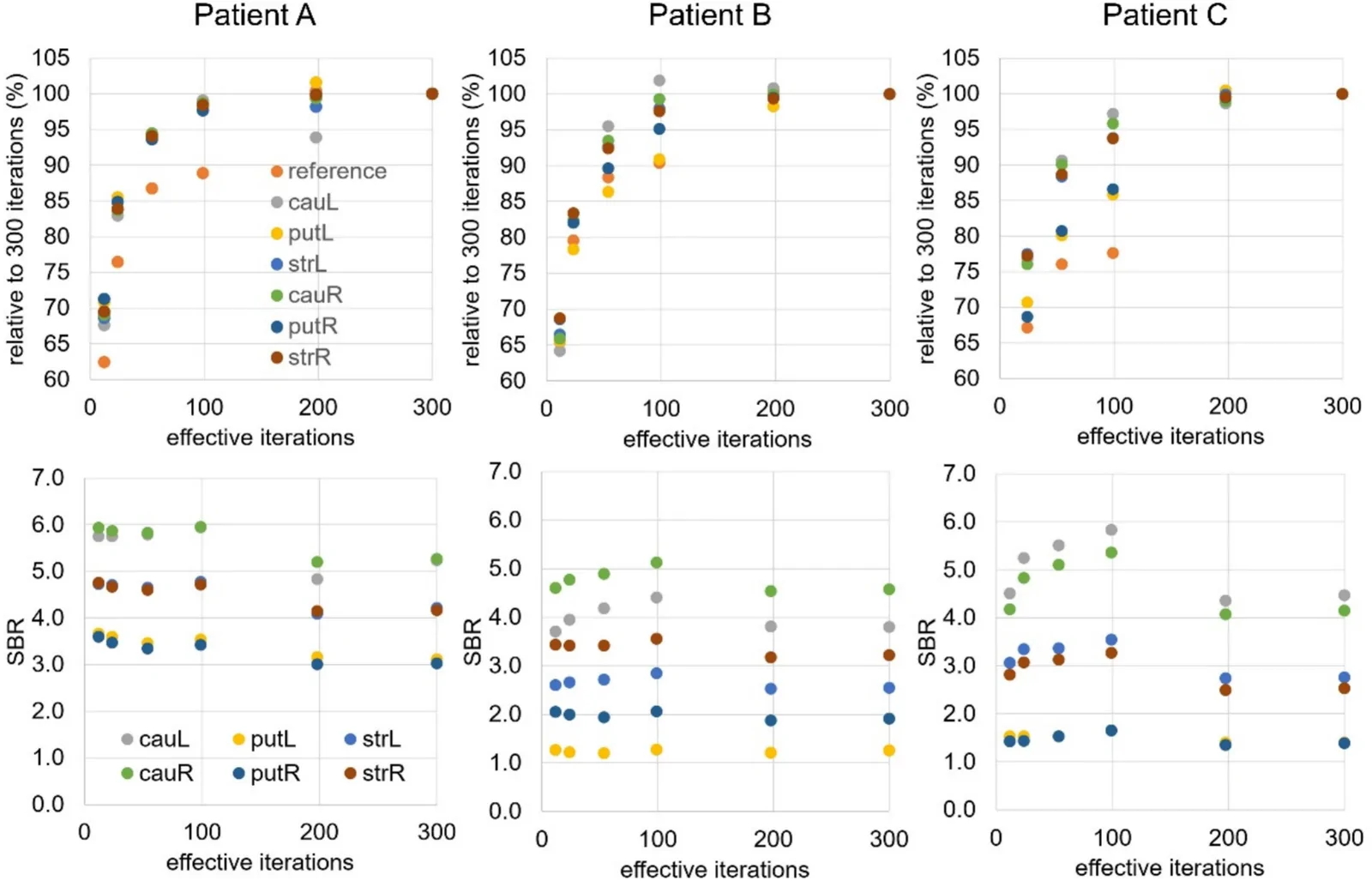
**Top:** Uptake in the reference region, left (L) and right (R) caudate nucleus (cau), putamen (put) and whole striatum (str) in the 3 patients from Fig. 2 as a function of the effective number of iterations. The reference region is characterized by the mean value of all voxels in the reference region, the striatal regions-of-interest are characterized by the mean value of the hottest voxels as described in the text. The uptake was scaled to the uptake at 300 effective iterations, separately for each region-of-interest. **Bottom:** Specific [^123^I]FP-CIT binding ratios (SBR) as a function of the effective number of iterations.

The results of the semi-quantitative analysis of these cases are shown in Fig. 3. Iterative reconstruction with 100 or less effective iterations resulted in considerable underestimation of the regional [^123^I]FP-CIT uptake, most pronounced in the extrastriatal reference region. This resulted in considerable overestimation of the [^123^I]FP-CIT SBR in the striatum and its subregions with 100 or less effective iterations compared to 300 effective iterations. SBR differences between 200 and 300 effective iterations were small.

### 3.2 Semi-quantitative analysis

**Fig. 4:**
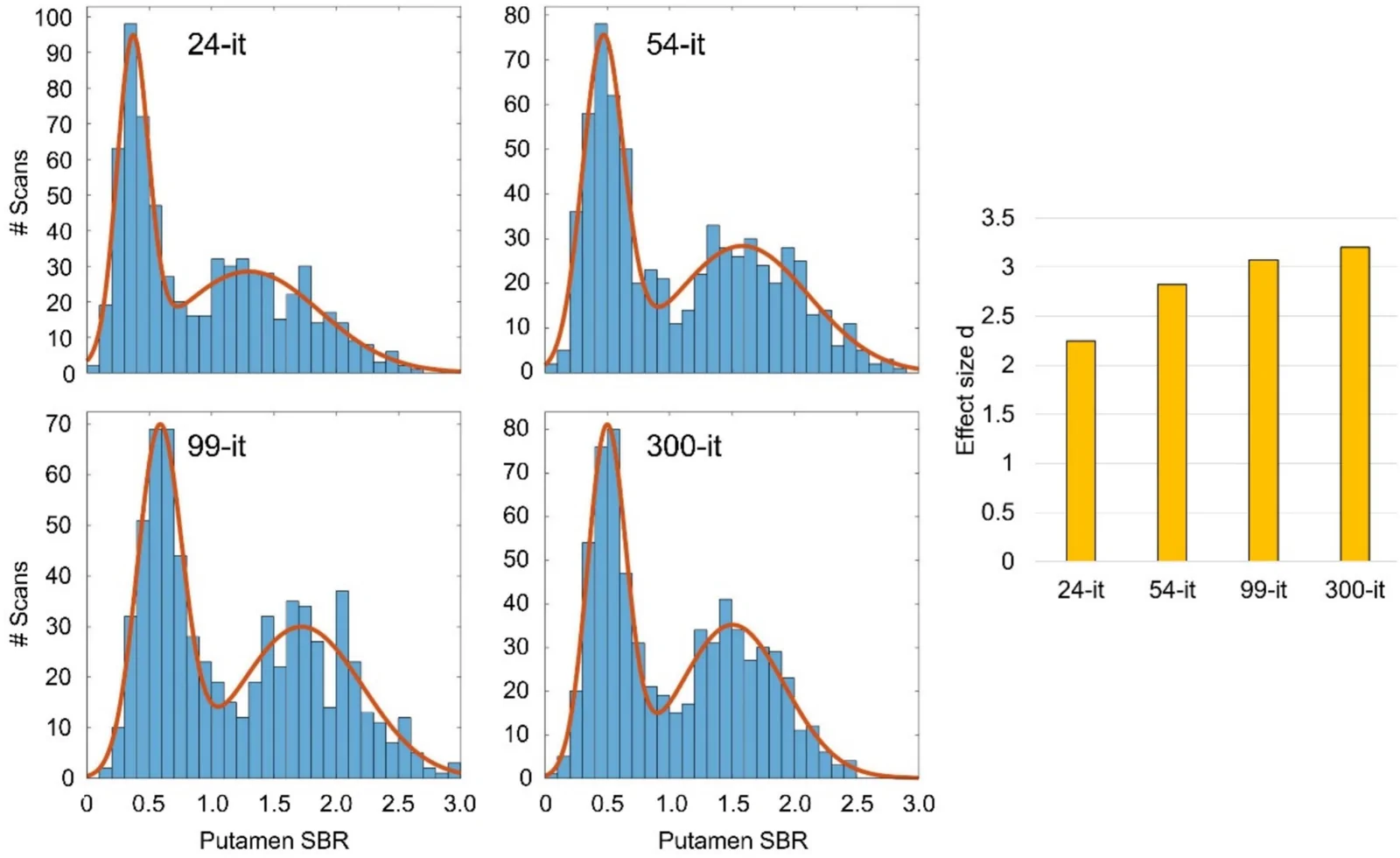
**Left:** Histograms of the putamen SBR (minimum of both hemispheres, n=671). The Gaussian mixture model fit is indicated by the red line. **Right:** Effect size *d* of the distance between the two Gaussian functions (according to Eq (2)). The remaining fit parameters are given in Supplementary Table 1.

Histograms of the putamen SBR (minimum of both hemispheres) across the 671 included patients are shown in Fig. 4 together with the Gaussian mixture model fit. The effect size d of the difference between reduced and normal cases increased with the effective number of iterations: from 2.250 at it-24 to 2.821 at it-54 to 3.072 at it-99 and to 3.202 at it-300.

### 3.3 Visual reading

#### 3.3.1 Binary majority vote

The binary categorization as normal or reduced based on the majority vote across all readers and both reading sessions did not differ between it-24 and it-300: 41 (57%) of the 72 cases included in the visual reading were categorized as reduced, 31 (43%) cases were categorized as normal.

#### 3.3.2 Repeated measure ANOVA of the Likert 6-score

**Fig. 5:**
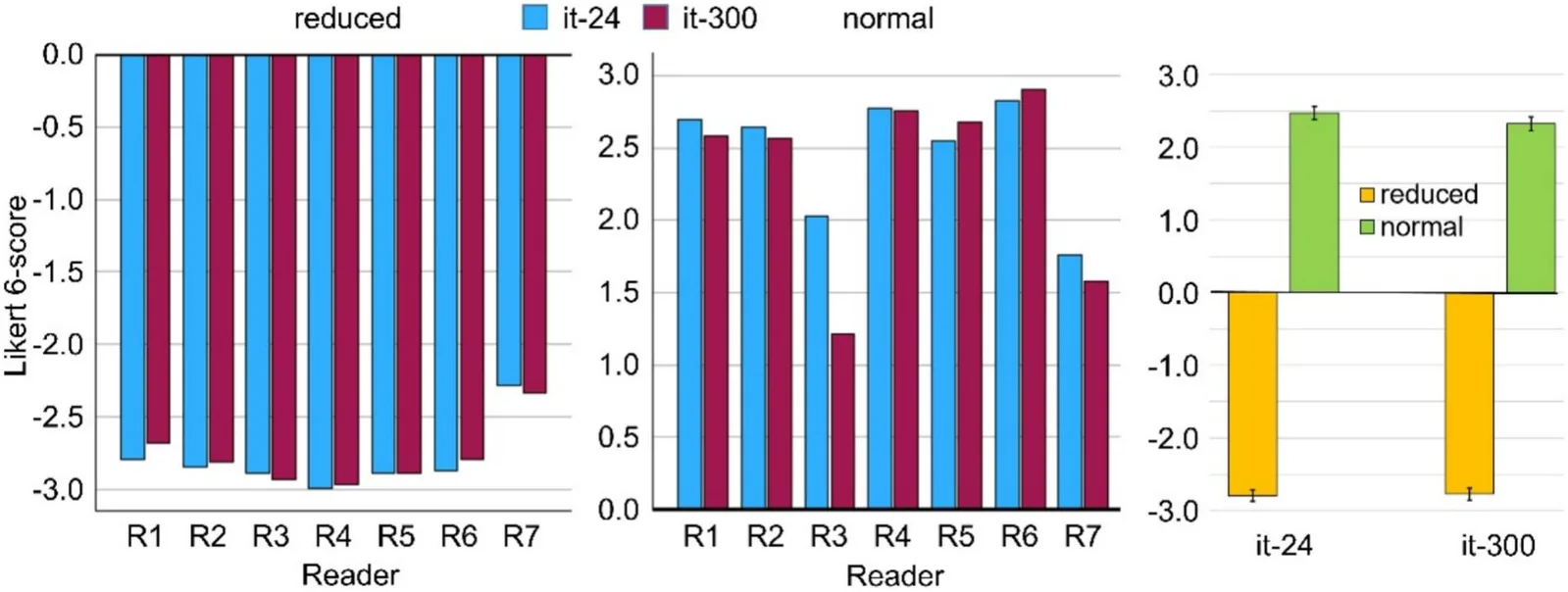
Repeated measure ANOVA of the visual Likert 6-score with reconstruction setting (it-24, it-300), reader (n = 7) and session (n = 2) as within-subject factors and the binary majority vote (reduced, normal) as between-subjects factor. The figure shows the estimated marginal mean of the Likert 6-score across both reading sessions, separately in reduced (left) and normal cases (middle). The estimated marginal means of the Likert 6-score across all readers and both reading sessions are shown on the right. The error bars represent the standard error.

The repeated measure ANOVA revealed reconstruction setting (p = 0.006) and reader (p < 0.001) but not session (p = 0.407) to have a significant effect on the Likert 6-score (Fig. 5). The diagnostic category (reduced, normal) x reconstruction setting interaction factor also had a significant effect (p < 0.001): the Likert 6-score was larger for it-24 compared with it-300 in normal cases (2.468 versus 2.325), whereas it was vey similar in both reconstruction settings in reduced cases (-2.793 versus -2.770, Fig. 5). Two of the inexperienced readers (R3 and R7, Fig. 5) scored with lower confidence than the experienced reader (R1, smaller abs(Likert 6-score)). The Likert 6-scores of the remaining inexperienced readers (R2 and R4-R6, Fig. 5) were similar to the Likert 6-scores of the experienced reader.

#### 3.3.3 Within- and between-readers agreement

**Fig. 6:**
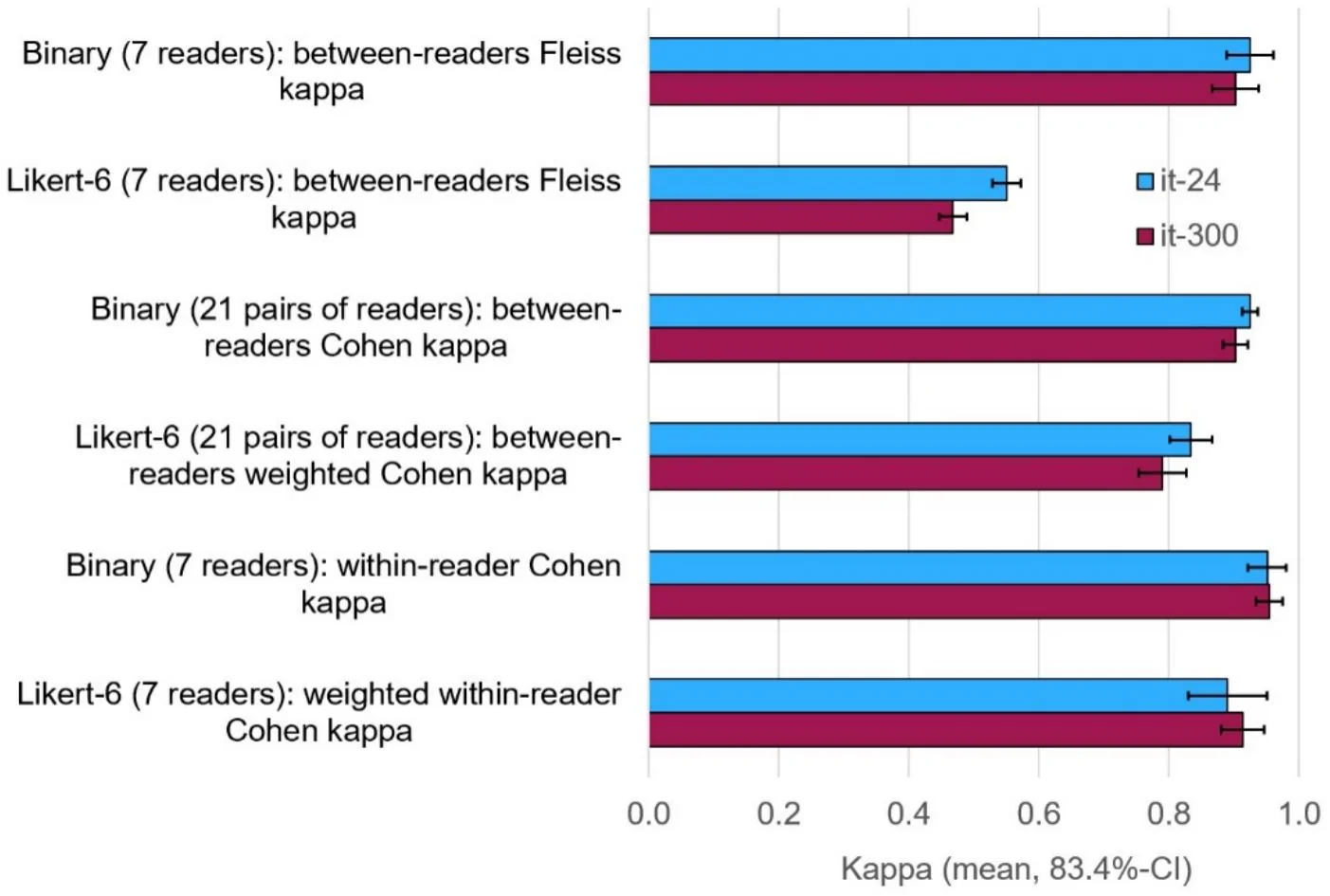
Within- and between-readers agreement of the Likert 6-score and of the binary categorization (reduced, normal) derived from the Likert 6-score. Within-reader agreement was characterized by Cohen’s kappa (linearly weighted for the Likert 6-score, unweighted for the binary categorization). Agreement between a pair of readers was characterized by Cohen’s kappa (linearly weighted for the Likert 6-score). Agreement between all readers was characterized by Fleiss’ kappa. The errors bars represent the 83.4% confidence interval (CI). The difference between the reconstruction settings (it-24, it-300) was considered statistically significant if the corresponding 83.4%-CI did not overlap.

The results regarding within- and between-readers agreement are summarized in Fig. 6. Fleiss kappa indicated moderate between-readers agreement regarding the Likert 6-score, significantly higher for it-24 (kappa = 0.550, 83.4%-CI 0.528 – 0.572) compared with it-300 (0.468, 83.4%-CI 0.447 – 0.489, non-overlapping 83.4%-CI). Regarding the binary categorization (normal, reduced) derived from the Likert 6-score, Fleiss kappa indicated almost perfect between-readers agreement, without significant difference between it-24 and it-300 (Fig. 6).Within-reader agreement was almost perfect regarding both the Likert 6-score and the binary categorization, without significant difference between it-24 and it-300 (Fig. 6).

## 4 Discussion

The aim of this study was to evaluate the impact of the effective number of reconstruction iterations in [^123^I]FP-CIT SPECT using a triple-head SPECT system with general-purpose brain imaging MPH collimators.

The primary findings of the study are the following. First, iterative reconstruction with 100 or less effective iterations resulted in considerable underestimation of the regional [^123^I]FP-CIT uptake (Fig. 3), indicating that the iterative reconstruction had not yet converged. The effect was more pronounced in the extrastriatal reference region than in the striatum and its subregions (Fig. 3), as expected from the larger relative noise level associated with the lower count density in the reference region. This resulted in considerable overestimation of the [^123^I]FP-CIT SBR in the striatum and its subregions with 100 or less effective iterations compared to 300 effective iterations (Fig. 3).

The delay of convergence of the iterative reconstruction in the reference region due to relatively reduced count sensitivity towards the edges of the FOV is expected to be the more pronounced the more peripherally the reference regions is located. Thus, reference regions restricted to cortical brain regions, e.g. the occipital cortex [4], might be more affected than the reference region used in the current study comprising the whole brain parenchyma (gray and white matter) without striata, thalamus, brainstem and cerebellum [27] (Fig. 1).

In line with this, consistent delineation of [^123^I]FP-CIT uptake in the scalp required at least 200 effective iterations (Fig. 2). The scalp is located most peripherally and, therefore, is most affected by the decrease of the MPH count sensitivity towards the edges of the FOV.

Second, increasing the number of effective iterations strongly improved the spatial resolution in the reconstructed images (Fig. 2). Clear separation between caudate nucleus and putamen in the reconstructed image required 100 or more effective iterations. With 300 effective iterations, the separation between caudate nucleus and putamen was almost PET-like (Fig. 2).

This was despite the fact that additional regularization was achieved for reconstruction with 200 or 300 effective iterations by increasing the image voxel size from 1.8 x 1.8. x 1.8 mm^3^ to 3.6 x 3.6. x 3.6 mm^3^ in order to avoid excessive noise amplification (Table 1). Still higher spatial resolution could be achieved by combining small image voxels and high iteration number with (deep learning-based) denoising (pre- and/or postreconstruction) [36].

There are early non-motor symptoms of Parkinson’s disease such as smell loss and rapid eye movement sleep and behavior disorder. High spatial resolution of MPH SPECT with 300 effective iterations could improve the sensitivity for the detection of mild nigrostriatal degeneration at prodromal (pre-motor) stages of the disease, when the loss of (unilateral) putaminal DAT is considerably below the 50% threshold for motor symptoms to occur [[37], [38], [39]].

Third, the effect size of the putamen SBR difference between reduced and normal cases according to the Gaussian mixture model increased with increasing the effective number of iterations (Fig. 4), suggesting that the diagnostic power of semi-quantitative analysis benefits from 300 effective iterations.

Fourth, 300 effective iterations resulted in slightly reduced confidence in the visual interpretation of [^123^I]FP-CIT SPECT compared to 24 effective iterations, more pronounced in normal than in reduced cases (Fig. 5). This could be explained by higher noise levels at 300 iterations (Fig. 2). The increase in noise with the increasing number of effective iterations was most pronounced in extrastriatal brain regions due to their relatively low count density (Fig. 2). However, a visible increase in noise levels was also seen in the striatum and its subregions, including the putamen (Fig. 2, patient A). A noisier appearance of normal putaminal signals is expected to result in reduced confidence in identifying normal [^123^I]FP-CIT SPECT images. However, the reduction in confidence when identifying normal cases was small (from 2.468 to 2.325 on the 6-point Likert scale ranging from -3 = clearly reduced to +3 = clearly normal). In clinical practice, [^123^I]FP-CIT SPECT interpretation is generally limited to distinguishing between a parkinsonian-like reduction in striatal signal, which indicates nigrostriatal degeneration, and a normal striatal signal, which indicates a secondary parkinsonian syndrome. It is unlikely that the observed reduction in confidence in identifying normal cases at 300 effective iterations leads to an increased rate of false-positive visual interpretations in clinical practice.

Fifth, between-readers agreement regarding the Likert 6-score as measured by Fleiss’ kappa was significantly smaller with 300 effective iterations compared with 24 effective iterations (Fig. 6), in line with lower reader confidence at 300 effective iterations. Between-readers agreement regarding the binary categorization of striatal [^123^I]FP-CIT uptake did not differ significantly between 300 and 24 effective iterations. Within-reader agreement also did not differ between 300 and 24 effective iterations, neither regarding the Likert 6-score nor regarding binary categorization.

Based on these findings, we recommend to use 300 effective iterations with the Tera-Tomo Monte Carlo simulation engine when [^123^I]FP-CIT SPECT is performed with a Mediso AnyScan Trio triple-head SPECT camera equipped with the second generation MPH brain collimators (full description of the recommended it-300 reconstruction setting is given in Table 1). Readers should be specifically trained for this reconstruction setting in order to avoid loss of reader confidence and increased between-readers variability.

The recommended number of 300 effective iterations for [^123^I]FP-CIT SPECT with MPH collimators is considerably larger than the number of approximately 100 effective iterations commonly recommended for (3D OSEM) iterative reconstruction in [^123^I]FP-CIT SPECT with conventional low-energy-high-resolution collimators [16,40]. This supports the hypothesis that the effective number of iterations required to avoid incomplete convergence of iterative reconstruction in [^123^I]FP-CIT SPECT is higher with MPH collimators compared to conventional low-energy-high-resolution collimators. However, incomplete convergence of iterative reconstruction primarily affects semi-quantitative analyses, the risk of false visual interpretation of [^123^I]FP-CIT SPECT in clinically uncertain parkinsonian syndromes is small.

The system count sensitivity profile of MPH SPECT can be widened in axial direction by increasing the total table displacement during helical acquisition. This is recommended when using the cerebellum as reference region in [^123^I]FP-CIT-SPECT [4]. However, this results in reduced count sensitivity in the peak of the sensitivity profile (where the striatum is located). Additionally, increasing the total table displacement during helical acquisition does not widen the sensitivity profile in radial direction.

A secondary finding of this study was that 4 of the 6 inexperienced readers scored very similar as the experienced reader (approximately 3,000 clinical cases in 15 years) (Fig. 5). This suggests that a 1h training can be sufficient to enable completely inexperienced readers to reliably interpret [^123^I]FP-CIT SPECT. The remaining 2 of the 6 inexperienced readers scored with lower confidence (Fig. 5), as expected.

Potential limitations of this study include the following. First, visual reading was based on a 12 mm thick slab image through the striatum [29,30]. The current findings with respect to visual reading may not be transferred without change to other reading methods, particularly when using thinner image slices (-> higher statistical noise level). The semi-quantitative analyses performed in this study used the full 3d SPECT images and, therefore, are not affected by any possible limitations of the slab images. Second, the current findings apply to [^123^I]FP-CIT SPECT only. They may not be transferred without change to other brain SPECT procedures. In particular, we hypothesize that spatially varying convergence of iterative MPH reconstruction could be more limiting in brain SPECT procedures, e.g. brain perfusion SPECT, in which the most relevant brain regions are located more peripherally rather than close to the center of the FOV. These brain SPECT procedures could also be more sensitive to minor off-center positioning of the brain, given the steep decline of the count sensitivity near the edges of the FOV.

## 5 Conclusions

When [^123^I]FP-CIT SPECT is performed with a Mediso AnyScan Trio triple-head SPECT camera equipped with second generation MPH brain collimators, 300 iterations of the Tera-Tomo Monte Carlo simulation engine seem to provide a good compromise between spatial resolution, robustness and diagnostic power of semi-quantitative SBR analyses on one side versus statistical noise and between-readers variability of the visual interpretation on the other side. In order to avoid relevant loss of between-readers stability, readers should be specifically trained for reading [^123^I]FP-CIT MPH SPECT images reconstructed with 300 iterations.

**Ethics approval and consent to participate**Waiver of informed consent for the retrospective analysis of the clinical data was obtained from the ethics review board of the general medical council of the state of Hamburg, Germany. All procedures performed in this study were in accordance with the ethical standards of the ethics review board of the general medical council of the state of Hamburg, Germany, and with the 1964 Helsinki declaration and its later amendments.

## CRediT authorship contribution statement

**Loreen Jedid:** Writing – original draft, Visualization, Validation, Methodology, Investigation, Formal analysis, Conceptualization. **Thomas Buddenkotte:** Writing – review & editing, Validation, Software, Methodology, Investigation, Formal analysis, Conceptualization. **Ivayla Apostolova:** Writing – review & editing, Validation, Methodology, Investigation, Formal analysis, Data curation. **Laurin Bruer:** Writing – review & editing, Validation, Investigation, Formal analysis. **Erik Jámbor:** Writing – review & editing, Validation, Software, Methodology, Investigation, Formal analysis. **Susanne Klutmann:** Writing – review & editing, Validation, Resources, Project administration, Methodology, Investigation. **Melinda Szolikova:** Writing – review & editing, Project administration, Methodology, Investigation, Formal analysis, Conceptualization. **Attila Forgács:** Writing – review & editing, Validation, Project administration, Methodology, Investigation, Formal analysis, Conceptualization. **Svend Hvidsten:** Writing – review & editing, Methodology, Investigation, Conceptualization. **Ralph Buchert:** Writing – review & editing, Visualization, Validation, Supervision, Project administration, Methodology, Investigation, Formal analysis, Data curation, Conceptualization.

## Funding

No funding was received for this project.

## Declaration of competing interest

The authors declare the following financial interests/personal relationships which may be considered as potential competing interests: Erik Jámbor, Melinda Szolikova and Attila Forgács are employees of Mediso Ltd, Budapest, Hungary. Ralph Buchert received research support from Mediso Ltd. The research support was paid to the institution. The other authors declare that they have no competing interests..
