# Supplementary material for: Delayed convergence of iterative reconstruction in dopamine transporter SPECT with multiple-pinhole collimators
Source: Z Med Phys. 2025 Nov 5;36(3):381–9. doi: 10.1016/j.zemedi.2025.10.006 (PMC13589428; doi:10.1016/j.zemedi.2025.10.006)
Supplement: Supplementary file 1 [file mmc1.docx]

**Supplementary material**

**Delayed convergence of iterative reconstruction in dopamine transporter SPECT with multiple-pinhole collimators**

Loreen Jedid^a^, Thomas Buddenkotte^a^, Ivayla Apostolova^a^, Laurin Bruer^a^, Erik Jámbor^b^, Susanne Klutmann^a^, Melinda Szolikova^b^, Attila Forgács^b^, Svend Hvidsten^c^, Ralph Buchert^a^

^a^Department of Diagnostic and Interventional Radiology and Nuclear Medicine, University Medical Center Hamburg-Eppendorf, Martinistr. 52, 20246 Hamburg, Germany

^b^Mediso Ltd., Laborc utca 3, H-1037 Budapest, Hungary

^c^Department of Nuclear Medicine, Odense University Hospital, J. B. Winsløws Vej 4, 5000 Odense C, Denmark

**Supplementary Tab 1.** Gaussian mixture model analysis of the putamen SBR. Mean values *M_1_*, *M_2_* and standard deviations *SD_1_*, *SD_2_* of the two Gaussians according to Eq (1) in the manuscript, and effect size *d* of their distance according to Eq (2).

|  | Reduced | | Normal | | Effect size |
| --- | --- | --- | --- | --- | --- |
| Setting | *M1* | *SD1* | *M2* | *SD2* | *d* |
| it-24 | 0.367 | 0.125 | 1.299 | 0.572 | 2.250 |
| it-54 | 0.466 | 0.165 | 1.585 | 0.536 | 2.821 |
| it-99 | 0.584 | 0.180 | 1.723 | 0.492 | 3.072 |
| it-300 | 0.497 | 0.155 | 1.506 | 0.418 | 3.202 |

**Supplementary Fig. 1** System sensitivity profile in axial and radial direction of the triple-head general-purpose camera (AnyScan Trio S, Mediso Medical Imaging Systems, Budapest, Hungary) equipped with second-generation general-purpose brain MPH collimators in helical acquisition mode with 40 mm total table displacement (as in the current study) [1]. The radial sensitivity profile was measured at the maximum of the axial profile.

[1] Mathies F, Apostolova I, Dierck L, Jacobi J, Kuen K, Sauer M, Schenk M, Klutmann S, Forgacs A, Buchert R. Multiple-pinhole collimators improve intra- and between-rater agreement and the certainty of the visual interpretation in dopamine transporter SPECT. EJNMMI Res. 2022;12:51. doi:10.1186/s13550-022-00923-w.

**Supplementary Fig. 2** Reader training: 20 training cases. Side-by-side display of the tested reconstruction settings (it-24, it-300) was used only during the training.

**Supplementary Fig. 3** Reader training: the Likert 6-score was intended to characterize the reader’s confidence in the binary classification of a case as either “reduced” or “normal”. The score was not intended for semi-quantitative grading of the striatal signal (e.g., to characterize the severity of nigrostriatal degeneration). Thus, clear loss of the [^123^I]FP-CIT signal in the posterior putamen in one hemisphere should be scored -3 = clearly reduced.

**Supplementary Fig. 4** Reader training: a rather uniform reduction of the striatal signal with clear delineation of the complete bilateral striatum should be scored 3 = clearly normal. Possible explanations of uniform reduction include old age, drug interaction and dementia with Lewy bodies.

**Supplementary Fig. 5** Reader training: the readers were asked for “conservative” interpretation by favoring specificity (for the detection of nigrostriatal degeneration) over sensitivity. Thus, borderline cases should be scored 1 (more likely normal than reduced) rather than -1 (more likely reduced than normal).
